# Supplementary material for: Corporate social responsibility and employee performance in China’s manufacturing sector: Exploring the roles of altruistic values and organizational identification
Source: PLoS One. 2026 Feb 4;21(2):e0339484. doi: 10.1371/journal.pone.0339484 (PMC12871998; doi:10.1371/journal.pone.0339484)
Supplement: S1 Appendix — (DOCX) [file pone.0339484.s001.docx]

**Survey questionnaire used in this study**

| Perceived CSR 1 | My company is truthful and fare with its employees. |
| --- | --- |
| Perceived CSR 2 | My employer cares about employee health and safety matters. |
| Perceived CSR 3 | My company tries to protect the environment. |
| Perceived CSR 4 | My company is honest with customers. |
| Perceived CSR 5 | My company contribute to social activities of the local community. |
| Perceived CSR 6 | My company operates according to the law. |
| In-role performance 1 | This employee meets the formal performance requirements of the job. |
| In-role performance 2 | This employee efficiently completes all his/her assigned duties. |
| In-role performance 3 | This employee fails to perform essential duties of his/her job (reversed code). |
| Extra-role performance 1 | This employee volunteers to do tasks for the work group. |
| Extra-role performance 2 | This employee helps other group members with their work. |
| Extra-role performance 3 | This employee helps to orient new employee. |
| Organizational identification 1 | When someone criticizes this company, it feels like a personal insult. |
| Organizational identification 2 | I am pleased to be a part of this company. |
| Organizational identification 3 | I talk about my organization by saying ‘we’ rather than ‘they’. |
| Organizational identification 4 | This company’s success is my success. |
| Organizational identification 5 | I am engaged in the growth of the company. |
| Altruistic values 1 | I volunteer for a charity. |
| Altruistic values 2 | I donate goods or clothes to people in need. |
| Altruistic values 3 | I offer seat on a public vehicle. |
| Altruistic values 4 | I help neighbors take care of people or pets. |
