# Supplementary material for: Corporate social responsibility and employee performance in China’s manufacturing sector: Exploring the roles of altruistic values and organizational identification
Source: PLoS One. 2026 Feb 4;21(2):e0339484. doi: 10.1371/journal.pone.0339484 (PMC12871998; doi:10.1371/journal.pone.0339484)
Supplement: Attachment — Submitted filename: to reviewers point ny point.doc [file pone.0339484.s002.doc]

**Dear Editor,**

**We sincerely appreciate your constructive feedback. The manuscript has been carefully revised with my supervisor, and we believe it now meets the journal's standards. Kindly review the updated version and advise if additional changes are needed.
Best regards,**

**The underlined contents are my responses to the your and reviewers' comments.**

PONE-D-25-18386
Corporate Social Responsibility and Employee Performance in China's Manufacturing Sector: Exploring the Roles of Altruistic Values and Organizational Identity
PLOS ONE

Dear Dr. fu,

Thank you for submitting your manuscript to PLOS ONE. After careful consideration, we have decided that your manuscript does not meet our criteria for publication and must therefore be rejected.

==============================

While your study addresses an important and timely topic, both reviewers identified several critical issues that significantly impact the manuscript’s suitability for publication. The primary concerns include:

1. The manuscript does not provide a robust theoretical framework or adequately integrate theory into the hypotheses and discussion. Both reviewers highlighted the **absence of a robust theoretical framework**. This undermines the study’s ability to make meaningful theoretical contributions.

**My reply: I have supplemented the theoretical framework, which can be found in the 'Theoretical Foundation: Social Identity Theory (SIT)' section of the INTRODUCTION.**

2.**The manuscript lacks a dedicated literature review section**, **which is essential for building a strong conceptual foundation.** **Hypotheses are introduced without sufficient theoretical grounding or critical analysis of prior studies**. This omission makes it difficult to justify the research gap and the rationale for the study. Without a clear explanation of how this paper advances existing knowledge, its value to the academic community remains uncertain.

**My reply: In response to the reviewers' suggestions, I have expanded the literature review by adding three dedicated sections addressing each research hypothesis: (1) Perceived CSR and Employee Performance, (2) The Mediating Role of Organizational Identification, and (3) The Moderating Role of Altruistic Values.**

==============================

**Comments to the Author**

1. Is the manuscript technically sound, and do the data support the conclusions?

The manuscript must describe a technically sound piece of scientific research with data that supports the conclusions. Experiments must have been conducted rigorously, with appropriate controls, replication, and sample sizes. The conclusions must be drawn appropriately based on the data presented.

Reviewer #1: Yes

Reviewer #2: Partly

My reply: The Methods section has been comprehensively elaborated to include detailed descriptions of: (1) sampling procedures, (2) measurement instruments, and (3) data analysis approaches. These methodological choices were rigorously aligned with established practices in prior studies addressing similar research questions. The current design ensures full correspondence with the theoretical requirements of our hypotheses while maintaining scientific rigor."

2. Has the statistical analysis been performed appropriately and rigorously?

Reviewer #1: Yes

Reviewer #2: Yes

3. Have the authors made all data underlying the findings in their manuscript fully available?

The [PLOS Data policy](http://www.plosone.org/static/policies.action#sharing) requires authors to make all data underlying the findings described in their manuscript fully available without restriction, with rare exception (please refer to the Data Availability Statement in the manuscript PDF file). The data should be provided as part of the manuscript or its supporting information, or deposited to a public repository. For example, in addition to summary statistics, the data points behind means, medians and variance measures should be available. If there are restrictions on publicly sharing data—e.g. participant privacy or use of data from a third party—those must be specified.

Reviewer #1: Yes

Reviewer #2: Yes

4. Is the manuscript presented in an intelligible fashion and written in standard English?

PLOS ONE does not copyedit accepted manuscripts, so the language in submitted articles must be clear, correct, and unambiguous. Any typographical or grammatical errors should be corrected at revision, so please note any specific errors here.

Reviewer #1: Yes

Reviewer #2: No

My reply: The manuscript has undergone professional English language editing to ensure compliance with academic writing standards. However, as a non-native English speaker, I acknowledge potential linguistic limitations. Should further language polishing be required, please do not hesitate to notify me—I would be grateful for any specific suggestions to enhance clarity.

**To reviewer 1,**

**Thank you very much for your thoughtful and constructive comments. I have carefully reviewed all of your suggestions, which have significantly improved both the quality of this manuscript and my academic writing skills. I sincerely appreciate your time and expertise.**

**In response to your feedback, I have thoroughly revised the manuscript. I would be most grateful if you could review these changes and provide any additional guidance you may have.**

**Once again, please accept my deepest gratitude for your valuable contribution to this work.**

.Review Comments to the Author

Please use the space provided to explain your answers to the questions above. You may also include additional comments for the author, including concerns about dual publication, research ethics, or publication ethics. (Please upload your review as an attachment if it exceeds 20,000 characters

Reviewer #1: Thank you very much for sharing this work with me to review. It is an interesting work that discusses the mediating role of organizational identity in the relationship between perceived CSR and employee performance and the moderating effect of altruistic values on this association. Although the work is timely and essential, I have some serious concerns, especially about the missing literature review and theoretical support for the job. I have the following suggestions:
1. The abstract is well written but needs some clarification on the study's theoretical and practical contributions.

**My reply: In accordance with your suggestion, the abstract has been comprehensively revised to better highlight both the theoretical and practical contributions of this study. The updated version now more clearly articulates:**

**These findings support the applicability of social identity theory (SIT) in explaining the CSR–employee work engagement relationship within the Chinese corporate context, while also identifying altruistic values as boundary conditions for this theoretical framework. This study contributes to the current understanding by highlighting the psychological mechanisms that link perceived CSR and employee behavior in Chinese cultural context.**

**2. Introduction: The first paragraph mentioned CSR as the voluntary adoption and government pressure of policies and practices that focus on social welfare, which is right, but not always the reason for organizations choosing CSR. Organizations are also pressured or forced by national/global rules and regulations, policies, competition, and global trends to adopt CSR. Missing that perspective is critical to developing a robust argument. The introduction needs a re-arrangement and a critical review to present ideas coherently.**
• **First, the context of the study (Chinese firms clearly mentioning larger firms, SMEs, public or private sector firms) should be mentioned explaining why this population is important and how the outcome variable (employee performance) is critical in this context. Transitioning to the second part which is, how outcome variable could improve (predicting variables)?**
• **Second, predicting variables should be introduced and discussed critically step by step starting from IVs and transitioning to mediator/s and moderators. This should outcome with a clear research question or research objectives of this study.**
• **Third, to address the proposed research question/objectives, a research gap paragraph should follow. The current research gap section is weakly developed using only six studies from different contexts (mostly older than 5 years), since this study is focused on Chinese firms, and there is a heap of literature on CSR activities in Chinese firms. The research gap section could benefit from a more in-depth analysis by critically discussing what and how past studies have examined about CSR, its predictors, outcomes and larger consequences proposing what is missing and why this study is needed.**
• **Fourth, a critical discussion on contributions of this study should be added by including theoretical approach selected (social identity theory), and logical explanations on how this study addresses the mentioned research gaps (including theoretical, methodological, or analysis techniques making this study unique from past studies).**
• A brief description of the remaining part of the paper must be provided for a reader flow.

**My reply: Following your suggestions, the Introduction section has been substantially revised. Given the extensive modifications made, I am unable to present all changes here comprehensively.**

3. **Literature Review: Literature review, including theoretical support, variable definitions, critical analysis of past studies on the chosen constructs, what has been done previously, and what the outcomes of past studies are missing. By saying that, I mean that the whole section of the literature review and hypothesis proposition is missing, which is critical for developing a sound basis for the proposed hypotheses. Mentioning hypotheses at the end of the introduction section is not recommended. There must be several sub-sections under the Literature review section such as:**
• **Theoretical support/foundation: Social identity theory should be explained (what is this theory, what are its elements/factors, prepositions) and how this study’s research model is linked to the social identity theory.**
• **Variable section according to proposed hypothesis including brief definitions of the constructs and then a critical discussion on what previous studies have said about the proposed relationships in this study. This should not only include arguments in support of the proposed relations but also critically reporting contradictory views from past studies.
• Each hypothesis section should also integrate social identity theory critically with the latest evidence (citations).
• A research model diagram would improve the presentation of the proposed study including hypothesis proposed.**

**My reply：I have comprehensively revised the entire Literature Review section to: (1) enhance the theoretical framework presentation, (2) incorporate targeted literature citations supporting each research hypothesis, and (3) develop a conceptual research model diagram. Given the substantial scope of these modifications, it would be impractical to enumerate all changes here, but they are clearly visible in the reviesed manuscript.**

4. Materials and Methods:
• **“In the present study, perceived CSR, organizational identity, altruistic values, in-role performance, and extra-role performance were 0.80, 0.84, 0.85, 0.79, and 0.72, respectively” this sentence is missing some clarification on what these values are.**
• **While the study offers valuable insights, I recommend the authors provide a brief clarification on the rationale for selecting Chinese firms as the data source, given that all authors are affiliated with Thai institutions. Clarifying the contextual relevance and data access arrangements would strengthen methodological transparency and enhance the study's credibility. This addition would also help readers better understand the applicability and generalizability of the findings.**

**My reply：I have made the following key improvements: (1) enhanced the documentation of measurement tools’ reliability and validity, including explicit reporting of Composite Reliability (CR) and Average Variance Extracted (AVE) values; and (2) strengthened the theoretical justification for focusing on Chinese manufacturing workers by adding contextual statements in the background section (e.g., "This study specifically focuses on the Chinese manufacturing industry, where collectivist values and the adoption of CSR practices are particularly prominent"). This sample selection reflects both methodological considerations (given my limited access to Thai industrial populations while studying abroad) and theoretical alignment with the research objectives.**

5. Results:
• **The study utilizes valuable dyadic data from supervisors and subordinates, but the analysis appears to treat these responses independently. Given the matched-pair design and nested structure (subordinates within supervisors), I recommend the authors consider applying multilevel modeling or paired-data analysis (e.g., ICC, paired t-tests) to more accurately reflect the data hierarchy and interdependence. This would enhance the rigor and interpretive value of the findings.**
• The study’s construct validation would benefit from further reliability and validity testing. In addition to Cronbach’s Alpha, it is advisable to assess composite reliability, convergent validity (e.g., AVE), and discriminant validity (e.g., Fornell-Larcker or item cross-loadings). Including exploratory or confirmatory factor analysis would also strengthen the credibility of the measurement model and support the theoretical structure of the constructs.
• While the study reports key regression outcomes such as R-squared values and standardized **coefficients, it would benefit from the inclusion of additional model evaluation metrics. Reporting appropriate model fit or effect size indicators, where applicable, would enhance the rigor and interpretive strength of the analysis. Such diagnostics help assess not just statistical significance, but also the practical relevance and robustness of the proposed relationships. Including these checks would provide a more comprehensive picture of model adequacy and strengthen the study’s methodological transparency.**

**My reply：Regarding the measurement instruments, I have supplemented the reliability and validity assessment by including Composite Reliability (CR) and Average Variance Extracted (AVE) values. For data analysis, multilevel modeling or paired-data analysis was not employed due to significant imbalance in the supervisor-subordinate structure - while some supervisors had as few as nine subordinates, others supervised over thirty employees. Finally, I have enhanced the results interpretation by incorporating effect size reporting, as demonstrated in this added statement: "The values of both effects exceed 0.30, indicating a strong effect according to conventional standards [58]."**

6. **Discussion and Implications: To further strengthen these sections, I recommend condensing some repetitive statements and adding brief summarizing remarks at the end of each subsection to clarify takeaways. Additionally, linking practical suggestions more directly to specific findings would help reinforce their empirical grounding. Overall, the discussion and implications are insightful, well-supported, and add clear value to both scholarly literature and managerial practice.**

**My reply：Following your suggestions, I have substantially revised the Discussion section to more prominently highlight both the theoretical contributions and practical implications of this study.**

**I hope my comments are constructive. Thank you very much.**

**To Reviewer #2,**

**Thank you very much for your insightful and constructive comments. After careful consideration, I fully agreed with your suggestions and have implemented extensive revisions to the manuscript accordingly. The revised version has now been resubmitted for your further evaluation. I would be most grateful for your continued guidance and feedback on the improved manuscript.**

**Once again, I sincerely appreciate your time and expertise in helping to enhance the quality of this work.**

Reviewer #2: I appreciate the authors efforts. The topic is interested but not properly executed. Please find my comments below:
1- The conceptualization is very weak. There is no any theoretical foundation.
2- The contribution is also questionable, gap is not properly defined, it is hard to find the rationale of study.
3- Literature review section is missing. The authors developed the hypotheses at the end of introduction without properly conducting the literature review.
4- Framework is very simple. There is no any novelty.
5- I would suggest the authors to read some articles of good journals to learn how to write a good paper.

6. PLOS authors have the option to publish the peer review history of their article ([what does this mean?](https://journals.plos.org/plosone/s/editorial-and-peer-review-process#loc-peer-review-history)). If published, this will include your full peer review and any attached files.

**Myply：Thank you very much for your valuable suggestions. In accordance with your comments, I have made extensive revisions to the manuscript. Given the substantial scope of these modifications, it would be impractical to enumerate all changes here. I would be most grateful if you could review the revised version and provide any additional feedback.**
