# Supplementary material for: Corporate social responsibility and employee performance in China’s manufacturing sector: Exploring the roles of altruistic values and organizational identification
Source: PLoS One. 2026 Feb 4;21(2):e0339484. doi: 10.1371/journal.pone.0339484 (PMC12871998; doi:10.1371/journal.pone.0339484)
Supplement: Attachment — Submitted filename: Response to Reviewers(1).doc [file pone.0339484.s003.doc]

**Dear Editor and reviewers**

I sincerely appreciate your constructive feedback. The manuscript has been carefully revised with my supervisor, and I believe it now meets the journal's standards. Kindly review the updated version and advise if additional changes are needed.

I have made major revisions to the manuscript, and I will strive to highlight the key changes.
Best regards

**To editor**

**1.Please ensure that your manuscript meets PLOS ONE's style requirements, including those for file naming. The PLOS ONE style templates can be found at**

**https://journals.plos.org/plosone/s/file?id=wjVg/PLOSOne_formatting_sample_main_body.pdf and**

[**https://journals.plos.org/plosone/s/file?id=ba62/PLOSOne_formatting_sample_title_authors_affiliations.pdf**](https://journals.plos.org/plosone/s/file?id=ba62/PLOSOne_formatting_sample_title_authors_affiliations.pdf)

**My reply:**

**Yes, I have made the revisions in accordance with the journal's requirements.**

2. **Please include a complete copy of PLOS’ questionnaire on inclusivity in global research in your revised manuscript. Our policy for research in this area aims to improve transparency in the reporting of research performed outside of researchers’ own country or community. The policy applies to researchers who have travelled to a different country to conduct research, research with Indigenous populations or their lands, and research on cultural artefacts. The questionnaire can also be requested at the journal’s discretion for any other submissions, even if these conditions are not met.  Please find more information on the policy and a link to download a blank copy of the questionnaire here: https://journals.plos.org/plosone/s/best-practices-in-research-reporting. Please upload a completed version of your questionnaire as Supporting Information when you resubmit your manuscript.**

**My reply:**

**Yes, I have carefully completed the PLOS Questionnaire on Inclusivity in Global Research. In addition, I have included a statement regarding the S1 File in the "Ethical Approval Statement" section of the manuscript, as shown below (see blue text):**

**3. Thank you for uploading your study's underlying data set. Unfortunately, the repository you have noted in your Data Availability statement does not qualify as an acceptable data repository according to PLOS's standards.**

**My reply:**

I have uploaded the raw data for this manuscript to Figshare. The data download link is as follows:

<https://figshare.com/s/5c295618c5fe18c42e04>

**4. When completing the data availability statement of the submission form, you indicated that you will make your data available on acceptance. We strongly recommend all authors decide on a data sharing plan before acceptance, as the process can be lengthy and hold up publication timelines. Please note that, though access restrictions are acceptable now, your entire data will need to be made freely accessible if your manuscript is accepted for publication. This policy applies to all data except where public deposition would breach compliance with the protocol approved by your research ethics board. If you are unable to adhere to our open data policy, please kindly revise your statement to explain your reasoning and we will seek the editor's input on an exemption. Please be assured that, once you have provided your new statement, the assessment of your exemption will not hold up the peer review process.**

**My reply:**

**I have uploaded the data to Figshare and added a "Data Availability Statement" in the "Introduction" section of the revised manuscript, as shown in the blue text：**

**5. If the reviewer comments include a recommendation to cite specific previously published works, please review and evaluate these publications to determine whether they are relevant and should be cited. There is no requirement to cite these works unless the editor has indicated otherwise.**

**My reply:**

**Noted. Thank you for the reminder!**

**Additional Editor Comments:**

**Both reviewers raised important points concerning the clarity of your introduction and literature review, the completeness and transparency of your methodological section (particularly the sampling procedure and validation of constructs), and the rigor of your results interpretation (e.g., more robust tests for mediation, clearer regression assumptions). You are also encouraged to improve figure captions, refine the language for clarity, and expand your discussion to better reflect limitations and alternative explanations.**

**My reply:**

**I have performed major revisions throughout the entire manuscript and have also polished the English language. The revised version presents a clearer line of reasoning, a more logical flow in the introduction, enhanced clarity in the methods section, and a more rigorous presentation of the research findings. In summary, the overall quality of the paper has been significantly improved. Should any further revisions be required, please do not hesitate to let me know.**

**Once again, I would like to express my sincere gratitude for the editor's comments and suggestions.**

**To reviewer 1,**

**Thank you very much for your thoughtful and constructive comments. I have carefully reviewed all of your suggestions, which have significantly improved both the quality of this manuscript and my academic writing skills. I sincerely appreciate your time and expertise.**

**In response to your feedback, I have thoroughly revised the manuscript. I would be most grateful if you could review these changes and provide any additional guidance you may have.**

**Once again, please accept my deepest gratitude for your valuable contribution to this work.**

.Review Comments to the Author

**1.Introduction – Streamlining and Emphasis on Novelty
The introduction provides a solid contextual background and clearly states the research hypotheses. However, certain literature review paragraphs are somewhat redundant or too detailed relative to the focus of the study.** **Condensing these sections would help direct the reader more quickly to the novelty of your approach.**

**My reply:**

**I have made major revisions to the "Introduction" section. I have streamlined this part by presenting the research background, theoretical foundation, and limitations of previous studies in a step-by-step manner, making the logic clearer and more fluid. The revised structure is as follows:**

**“CSR in China→the associations between CSR and employee outcomes→SIT can explain these associations→the content of SIT→literature evidence SIT notions on the link between perceived CST and employee performance→SIT implies the role of altruistic values→prior studies evidence the moderation of altruistic values→the gaps of the previous literature→the aim of the study →theoretical framework and hypotheses”**

**Due to the extensive revisions made to the "Introduction" section, the changes are too extensive to list individually. Please refer to the revised manuscript to see the updated version.**

**2. Methods – Completeness of Description
The methodological section is clear but could benefit from a fuller description of:
（1） The rationale for selecting specific variable transformations and the criteria used for model selection.
（2）The procedures adopted to check statistical assumptions (e.g., normality, homoscedasticity, absence of multicollinearity (VIFs are reported only in a note to Table 2).
Including these details will improve reproducibility and transparency.**

**My reply:**

（1）**I have added the rationale for selecting specific variable transformations as follows (see the text in blue):**

**（2）I have added the procedures to check statistical assumptions (e.g., normality, homoscedasticity, absence of multicollinearity) to Table 2 (see the text in blue).**

**Additionally, given the large sample size of this study, the Central Limit Theorem ensures that the sampling distributions of the estimated model parameters (such as β1 and β2) will approach normality, even if the error terms are not perfectly normally distributed. Therefore, no additional explanation regarding normality tests has been included in the revisions.**

**3. Results – Clarity and Integration with Hypotheses
Results are presented in a logical order, but in some cases the link between numerical outputs and the corresponding hypotheses could be made more explicit in the text. This would strengthen the narrative flow and help the reader follow how the evidence addresses each hypothesis.
Additionally, while figures are generally clear, in Figure 1 there is a typographical error (perceived not perceive CSR) and Figure 3 have axes or legends that are difficult to interpret without referring to the text. Also captions should be more self-contained.**

**My reply：**

**I have performed major revisions on the "Results" section to enhance its clarity and make it self-contained. For instance, key statistical measures have been incorporated into this section, as illustrated below (see text in blue)：**

<https://www.baidu.com/link?url=KniIAFMIHXghNKxhOmoGT3TUxUDOKn7oeha8Ikp6BjJ17fy0fbg3aB2lpTDgHdLsSRhLKOP1BJki7iXhSq_w1sy8ADIBfNvrJmOcbbUTg0HicdWGP3unHECm2zf7GlAvcfadOm98trZ2S_EBRWDhyq&wd=&eqid=d09289ef003a1bef0000000468dd184b>

[**⋯⋯**](https://www.baidu.com/link?url=KniIAFMIHXghNKxhOmoGT3TUxUDOKn7oeha8Ikp6BjJ17fy0fbg3aB2lpTDgHdLsSRhLKOP1BJki7iXhSq_w1sy8ADIBfNvrJmOcbbUTg0HicdWGP3unHECm2zf7GlAvcfadOm98trZ2S_EBRWDhyq&wd=&eqid=d09289ef003a1bef0000000468dd184b)

**Additionally, I have revised Figure 1 by adding the path from perceived CSR to employee performance (indicated by the blue line).**

**Finally, I have revised the text preceding and following Figure 2 to enhance the clarity and comprehensibility of the meaning conveyed by the figure (as shown in blue font).**

**4. Discussion – Critical Engagement
The discussion appropriately summarizes the main findings, but could be strengthened by:
• Providing a more explicit reflection on potential alternative explanations for the observed patterns.
• Expanding the implications of limitations, particularly in terms of the dataset’s spatial and temporal scope.
• Ensuring that policy or practical implications are clearly framed as either evidence-based or speculative, to avoid overstatement.**

**My reply：**

**• I have incorporated the following additions into the discussion section (as highlighted in blue) to provide a more explicit reflection on potential alternative interpretations of the observed results:**

In my view, including additional potential alternative explanations in the Discussion section offers a more natural presentation and clearer logical structure. Introducing these points in the "Limitations" or "Implications" sections would interrupt the coherence of the narrative.

**•I have incorporated the following content into the "Limitations" section (as highlighted in blue) to clarify the limitations of the sample.**

**• I have rewritten the "Implications" section to better align with the findings of this study and to refrain from overstatement. The specific content is as follows:**

 **5. Language and Formatting
The manuscript is overall well-written, but some sentences are long and could be simplified for clarity. Minor grammatical issues and small inconsistencies in reference formatting should be addressed with a final proofreading.**

**My reply：**

**I have polished the language throughout the entire manuscript, and I believe the overall quality of writing has been significantly improved.**

**Thank you again for your comments and suggestions. If any further revisions are needed, please let me know!**

**Once again, I would like to express my sincere gratitude for the reviewer 1's comments and suggestions.**

**To Reviewer #2,**

**Thank you very much for your insightful and constructive comments. After careful consideration, I fully agreed with your suggestions and have implemented extensive revisions to the manuscript accordingly. The revised version has now been resubmitted for your further evaluation. I would be most grateful for your continued guidance and feedback on the improved manuscript.**

**Once again, I sincerely appreciate your time and expertise in helping to enhance the quality of this work.**

**Reviewer #2: The paper exploits Social Identity Theory (SIT) to investigate how Corporate Social Responsibility (CSR) impacts employee performance when considering organizational identification as a mediator and altruistic values as a moderator.
However, in order to evaluate the presented results, further clarification and analysis are necessary. Furthermore, not all sections of the manuscript are written with sufficient detail to be accessible to non-specialists.
My overall assessment is that this paper shows potential but some of my remarks should be considered (with related adjustments) in order to proceed with the publication process.
Below, I provide a number of major and minor remarks.**

**Myply：**

**I have undertaken substantial revisions to the entire manuscript. The logical flow has been restructured to enhance its coherence and clarity. I believe the revised version represents a significant improvement in quality compared to the previous draft.**

**INTRODUCTION
The introduction positions the analysis consistently within the relevant literature but some adjustments are necessary to make the manuscript more intelligible.
The introduction should cover both the theoretical foundations and the related empirical contributions. In addition, it should provide a brief explanation of the objectives of the study and the overall structure of the paper. Ideally, one should be able to grasp the essence of the work by reading only the introduction and the conclusions. This is not the case here and I would therefore recommend including at least a summary of the main findings as well as a clearer description of the paper’s structure.
Other minor issues:
- “Of the numerous theoretical models employed to explain these relations, Social Identity Theory (SIT)” You are not actually referring to a formal economic model. I would rather describe this as a theoretical framework. Similarly, I would avoid referring to Figure 1 as a theoretical model.
- “Furthermore, this research contributes theoretical insights to the CSR literature by illuminating the processes that connect external CSR perceptions with internal value-based responses.” Not very clear.**

**Myply：**

**I have implemented major revisions to the "INTRODUCTION" section and restructured its logical flow to enhance the overall coherence of the paper. The revised structure is as follows:**

**“CSR in China→the associations between CSR and employee outcomes→SIT can explain these associations→the content of SIT→literature evidences SIT notions on the link between perceived CST and employee performance→SIT implies the role of altruistic values→prior studies evidence the moderation of altruistic values→the gaps of the previous literature→the aim of the study →theoretical framework and hypotheses”.**

**Thus, the introduction now provides a comprehensive overview of both the theoretical foundations and relevant empirical studies, making it more intelligible.**

**Additionally, I have revised the study objectives as follows (as indicated in blue):**

**Finally, regarding the suggestion to add the overall structure of the paper in the Introduction, I believe this is unnecessary. Given that the paper is not particularly lengthy, its organization is clear and the first-level headings are already prominently displayed. Including such a section would be redundant.**

**I have reworded the phrase "Of the numerous theoretical models employed to explain these relations, Social Identity Theory (SIT)" as follows (shown in blue):**

**Finally, I have removed the following sentence: "Furthermore, this research contributes theoretical insights to the CSR literature by illuminating the processes that connect external CSR perceptions with internal value-based responses." I have then rewritten the objectives of this study as follows (see text in blue):**

**METHODS
The sampling procedure is not explained. How were the enterprises selected? Without this information the results may not be generalizable to the target population. This clarification is essential in order to properly assess the validity of the manuscript’s findings.
- It would also be useful to provide a description of the measurement items, even if only in the annexes.
- The expression “deprived from a previous study” should be revised, as it appears to be incorrect.
- The subsection “Data Analysis” is too brief. Key information should be presented here rather than scattered across the results section, otherwise the subsection loses its purpose.**

**My reply：**

**The acquisition process of the sample is summarized in one sentence in this study (as shown in blue):**

**The measurement instruments used in this study are presented in the Appendix, as shown in the blue text.**

**Regarding the comment that "The expression 'deprived from a previous study' should be revised, as it appears to be incorrect," I have replaced "deprived" with "adapted" as shown in the blue text.**

**We have reworded the data processing methodology to make it more concise and clear, as shown in the blue text.**

**RESEARCH RESULT**
**Although the constructs show good measurement reliability, additional diagnostics are required before the results can be properly evaluated, particularly in light of the detected correlations among the constructs.
Major points:
- Harman’s single-factor test is an ex-post statistical check, not a method to anticipate common method bias. Moreover, this test is limited, as it does not account for the possibility that common method variance (CMV) may load onto multiple general factors. A more appropriate approach would be to conduct a Confirmatory Factor Analysis (CFA) including all items. In this specification an additional latent factor is introduced onto which all items load, in addition to their respective constructs. If this additional factor explains a substantial share of the variance (commonly more than 30%) common method bias is present. The decision to avoid using CFA should therefore be explained and properly justified.
Minor points:
- The regression assumptions should be explicitly verified. If this was done, it should at least be stated that they were confirmed (e.g., multicollinearity and homoscedasticity).
- In Table 2, the analytical steps are correct, but in the text it is reported that: “In Model 2, another independent variable, M, was included in the regression model alongside X as predictors.” Here, however, the mediator is the outcome. The steps should be explained with greater clarity in the text. It is not necessary to follow the variable nomenclature used by Namazi and Namazi (2016); it would be clearer to adopt the nomenclature actually employed in the analysis.
- An increase in the R^2 is not sufficient to establish a mediation effect.
- Why was a bootstrapping method not employed for the mediation analysis as well?
- The reasons why perceived CSR and altruistic values were mean-centered prior to analysis should be mentioned.
- Why did you not consider the SEM framework for your analysis?**

**My reply：**

**First, in the test for common method bias, I have additionally included the results of the single-factor measurement model as follows (see the text in blue):**

We have added a statement on homogeneity of variance in the note below Table 2 (as shown in blue font).

**Simultaneously, we have reformulated the method for testing mediating effects to enhance its clarity (as shown in the blue text).**

**Since R² is not sufficient to establish a mediation effect, I have removed all references to R-squared from the relevant sections (as shown in blue).**

**Since this study did not utilize the PROCESS plugin for SPSS, the bootstrapping method could not be employed to test the mediation effects.**

**In the revised manuscript, I have added a sentence stating the reasons for mean-centering perceived CSR and altruistic values prior to analysis (as shown in blue).**

**Finally, this study did not employ Structural Equation Modeling (SEM) to test the hypotheses for two reasons. First, using stepwise regression analysis allows for the verification of the three hypotheses individually, which better reflects the logical progression of the proposed research hypotheses. Second, SEM is less straightforward for controlling demographic variables such as gender, education level, and age.**

**DISCUSSION, IMPLICATIONS, LIMITATIONS AND CONCLUSIONS
These sections should be reconsidered in light of the requested revisions. At present, the claims are not, in the reviewer’s view, sufficiently well supported.**

**My reply：**

**I have made major revisions, and in some cases completely rewritten, the DISCUSSION, IMPLICATIONS, LIMITATIONS, AND CONCLUSIONS sections. This was done to enhance their clarity and rigor, and to ensure they are better aligned with the INTRODUCTION. The results of these revisions can be seen in the revised manuscript.**

**Thank you once again for your valuable feedback. I am prepared to make any additional modifications as needed.**
