# Supplementary material for: Corporate social responsibility and employee performance in China’s manufacturing sector: Exploring the roles of altruistic values and organizational identification
Source: PLoS One. 2026 Feb 4;21(2):e0339484. doi: 10.1371/journal.pone.0339484 (PMC12871998; doi:10.1371/journal.pone.0339484)
Supplement: Attachment — Submitted filename: respons to reviewers.doc [file pone.0339484.s004.doc]

Dear Editors and Reviewers,

Thank you very much for your comments! I have carefully read your evaluations and suggestions, thoroughly reviewed the manuscript, and made revisions one by one in accordance with your requirements. Please feel free to inform me if there are any inappropriate parts, and I will revise them diligently again. Thank you so much!

**To editor**

**Editor’s comment 1: Expand the Introduction with a brief summary of the study’s main findings and structure.**

**My reply:**

I have added a brief summary of the study’s main findings and structure in the "Introduction" section as follows (see the content in blue).

This study demonstrates that the relationship between perceived CSR and employee performance is partially mediated by organizational identification, and that altruistic values negatively moderate the link between perceived CSR and organizational identification. It enriches SIT within the Chinese cultural context and contributes to improving management practices in Chinese enterprises. The remainder of the study is organized as follows: (1) Methods (sampling and measures); (2) Research results (mediation and moderation analyses); (3) Discussion; (4) Implications; (5) Limitations; (6) Conclusions; and (7) Appendix (containing the measurement scales).

**Editor’s comment 2: In the Limitations section, explicitly note that the sample was non-probabilistic and discuss the implications for generalizability.**

**My reply:**

To highlight the limitations of non-probabilistic sampling, I have added the following content in the "Limitations" section (see the content in blue).

Finally, this study employed a non-probabilistic sampling method, drawing its sample exclusively from manufacturing firms in Sichuan Province. This approach may thereby constrain the generalizability of the findings to other regions or industries. To enhance the external validity of future research, it would be beneficial to employ probability sampling techniques across more diverse geographical contexts..

**Editor’s comment 3: Clarify that Harman’s test was conducted as an ex-post check for common method bias (see** **Podsakoff et al., 2003).**

**My reply:**

I have added the following content in the "Common Method Variance "section (see the content in blue).

First, Harman's single-factor test was conducted as an ex-post check[45]**.**

I have also added the literature you provided i in the references (see the content in blue):

45.	Podsakoff PM, MacKenzie SB, Lee J-Y, Podsakoff NPJJoap. Common method biases in behavioral research: a critical review of the literature and recommended remedies. Journal of applied psychology. 2003;88(5):879.

**Editor’s comment 4: Conduct a final proofreading to ensure smooth, concise language.**

**My reply:**

I have carefully read through the entire manuscript and revised every detail that required modification. I firmly believe that the quality of my manuscript has been greatly improved and now meets the publication standards of PLOS ONE.

Finally, I have carefully reviewed all the literature and found no retracted papers. All the references are from truly reliable sources.

**To reviewer 1**

**Comment 1: A more explicit consideration of** **alternative explanations and the broader theoretical implications would strengthen the discussion.**

**My reply:**

I have appropriately added content related to alternative explanations in the "Discussion" section (see the content in blue):

For example, one study found that organizational trust and job satisfaction can mediate the relationship between perceived CSR and employee performance[65]. Another study demonstrated that work meaningfulness and organizational pride sequentially mediate the link between perceived CSR and job performance among Chinese employees[66]. In addition, another potential explanatory mechanism deserves consideration. Within China's cultural context, where authoritarian norms are prevalent [67], employees' work performance and CSR compliance may result more from deference and loyalty to management than from genuine endorsement of CSR or organizational identification. In summary, beyond the mechanism of organizational identification grounded in SIT, other mechanisms may also partially account for the relationship between perceived CSR and employee performance.

**Comment 2: The conclusion could more clearly articulate the study’s limitations (I suggest to move the limitation section in the conclusions) and outline possible directions for future research.
My reply:**

I have carefully revised the "Limitations" section and clearly stated the potential future research directions (see the content in blue).

This study has three notable limitations that warrant attention. First, it employs cross-sectional data, which allows only correlational, not causal, conclusions. Future research should incorporate longitudinal follow-ups with participants to provide more robust evidence supporting the relationships among perceived CSR, organizational identification, and employee performance. Second, the present study examines the associations between perceived CSR and employee performance using an individual-level model, thereby overlooking potential organization-level influences. Future research would benefit from exploring employees' responses to CSR within a multilevel framework. Finally, this study employed a non-probabilistic sampling method, drawing its sample exclusively from manufacturing firms in Sichuan Province. This approach may thereby constrain the generalizability of the findings to other regions or industries. To enhance the external validity of future research, it would be beneficial to employ probability sampling techniques across more diverse geographical contexts.

**Comment 3: Finally, a careful language proofreading is advised to smooth a few long or complex sentences.
My reply:**

I have carefully read every sentence of the manuscript and conducted a careful language proofreading.

**To reviewer 2**

**Comment 1: The introduction should cover both the theoretical foundations and the related empirical contributions. In addition, it should provide a brief explanation of the objectives of the study and the overall structure of the paper. Ideally, one should be able to grasp the essence of the work by reading only the introduction and the conclusions. This is not the case here and I would therefore recommend including at least a summary of the main findings as well as a clearer description of the paper’s structure.”
My reply:**

I have carefully read every sentence of the manuscript and conducted a careful language proofreading. Many minor revisions cannot be listed one by one here. In short, I have made every effort to improve the quality of the manuscript.

Meanwhile, I have provided a brief explanation of the study’s objectives and the overall structure of the paper; please refer to the following content (in blue):

This study demonstrates that the relationship between perceived CSR and employee performance is partially mediated by organizational identification, and that altruistic values negatively moderate the link between perceived CSR and organizational identification. It enriches SIT within the Chinese cultural context and contributes to improving management practices in Chinese enterprises. The remainder of the study is organized as follows: (1) Methods (sampling and measures); (2) Research results (mediation and moderation analyses); (3) Discussion; (4) Implications; (5) Limitations; (6) Conclusions; and (7) Appendix (containing the measurement scales).

**Comment 2: Typo: “For example, an study of** **multinational financial corporations revealed”**

**My reply:**

I have corrected this minor mistake, as shown below (in blue font):

For example, an empirical study of multinational financial corporations revealed that community-oriented CSR positively influenced employees' in-role performance,

**Comment 3: I would avoid mentioning literature that highlights how organizational identification moderates the impact of perceived CSR on task performance and helping behavior in manufacturing environments when discussing its mediating effect (p. 4).**

**My reply:**

I have deleted this paragraph and added the corresponding content as follows (see blue font):

Quantitative studies based on SIT revealed that organizational identification could mediate the relationship between CSR and employee outcomes. A study in Western cultural contexts revealed that perceived CSR enhanced organizational identification, which in turn improved employee adjustment and job performance[24]. Organizational identification and job satisfaction were found to serially mediate the link between CSR and work performance among Korean employees [25], while organizational identification and commitment channeled CSR's effects on performance and turnover intention in Saudi workers[26]. Consistent with these findings, a Chinese study found that organizational identification mediated the relationship between perceived CSR and employee behaviors, including turnover intention, helping behavior, and in-role performance[1].

**Comment 4: Purposive sampling is not a probabilistic method, therefore it would be important to explain how the enterprises were selected and to acknowledge the limitations of this sampling approach. The results and conclusions should be discussed in light of these limitations.**

**My reply:**

First, the following content (see blue font) has been added to the "Sampling Procedure" section to clarify the specific details of the sampling:

The study employed a purposive sampling approach to identify eligible enterprises based on predefined criteria: (1) operation in the manufacturing sector, (2) documented implementation of CSR initiatives, and (3) expressed willingness to participate in the survey. Subsequently, a convenience sampling method was used to recruit accessible frontline supervisors—each required to have at least ten direct subordinates and agreed to cooperate—along with their respective teams within these organizations. The sample comprised three state-owned and four privately owned firms from diverse sectors, including machinery, food production, household appliances, and apparel. The employees surveyed were exclusively front-line workers responsible for production tasks, quality control, sales, marketing, and related activities. We contacted the direct supervisors of these frontline employees across the companies and requested their assistance in facilitating the investigation. This approach ensured the confidentiality of the participants[37].

Second, I have revised the corresponding content in the "Limitations" section to highlight the impact of non-probability sampling on the research results (see blue font):

Finally, this study employed a non-probabilistic sampling method, drawing its sample exclusively from manufacturing firms in Sichuan Province. This approach may thereby constrain the generalizability of the findings to other regions or industries. To enhance the external validity of future research, it would be beneficial to employ probability sampling techniques across more diverse geographical context..

**Comment 5: Include a reference to the appendix for the listing of measurement instruments (a footnote would also suffice).**

**My reply:**

I have added "see Appendix 1" to the "Measurement Instruments" section, as shown in the following content (see blue font):

The measure of CSR is adapted from a prior study [38]. It encompasses six key items (see Appendix 1):

A 5-item scale was used to measure organizational identification [39] (see Appendix 1).

Employee performance was measured using a six-item scale adapted from two previous studies [43, 44] (see Appendix 1).

**Comment 6:** **The expression “deprived from a previous study” should be revised, as it appears to be incorrect.**

**My reply:**

I have revised this improperly expressed part, as shown below (in blue font): One of the items was: donate clothes or goods to people in need. These items were adapted from previous studies[40-42].

**Comment 7:** **The subsection “Data Analysis” is too brief. Key information should be presented here rather than scattered across the results section, otherwise the subsection loses its purpose.**

**My reply:**

I have made appropriate revisions to the description of data processing to make it clearer (see blue font):

First, Harman's single-factor test was conducted as an ex-post check[45]**.** It revealed four factors with eigenvalues exceeding 1, with the first factor explaining 32.72% of the total variance—below the 40% threshold—indicating no significant common method bias in the survey data[46]. Second, confirmatory factor analysis for a single latent factor demonstrated poor model fit (RMSEA = 0.13, CFI = 0.56, TLI = 0.51, SRMR = 0.11). The common factor accounted for only 28.57% of the total variance on average (R² range: 0.13–0.44), which is well below the 50% threshold, thereby confirming the absence of common method bias.

**Comment 8: Harman’s single-factor test is an ex-post statistical check, not a method to anticipate common method bias. Claiming otherwise is simply incorrect.**

**My reply:**

I have added a sentence to the "Common Method Variance" section to emphasize "an ex-post statistical check" (see blue font).

First, Harman's single-factor test was conducted as an ex-post check[45]**.** It revealed four factors with eigenvalues exceeding 1, with the first factor explaining 32.72% of the total variance—below the 40% threshold—indicating no significant common method bias in the survey data[46]. Second, confirmatory factor analysis for a single latent factor demonstrated poor model fit (RMSEA = 0.13, CFI = 0.56, TLI = 0.51, SRMR = 0.11). The common factor accounted for only 28.57% of the total variance on average (R² range: 0.13–0.44), which is well below the 50% threshold, thereby confirming the absence of common method bias.

In addition, the following text has been added to the "Measurement Instruments" section (see the blue font).：

To minimize potential common method bias, all measurement items were carefully developed to be clear, simple, and concise, avoiding ambiguous or double-barreled statements. In addition, items for all constructs used specific and unambiguous wording to reduce context-induced mood effects and were further randomized in the questionnaire[45, 46].

**Comment 9: Regarding the CFA, it is necessary to report the total variance explained by the construct. I therefore recommend following the guidance in Podsakoff et al. (2003).
My reply:**

Finally, I have added a sentence to the "Common Method Variance" section to present the total variance explained by the construct (see blue font).

Second, confirmatory factor analysis for a single latent factor demonstrated poor model fit (RMSEA = 0.13, CFI = 0.56, TLI = 0.51, SRMR = 0.11). The common factor accounted for only 28.57% of the total variance on average (R² range: 0.13–0.44), which is well below the 50% threshold, thereby confirming the absence of common method bias.
**Comment 10: On page 13 (text in red), Model 2 and Model 3 appear to be described identically, although Table 2 clearly indicates that Model 2 is actually a regression of OI on CSR. This should be explained better.**

**My reply:**

I have carefully revised this part of the content to make the expression clearer (see the content in blue font):

Secondly, Model 2 revealed that perceived CSR had a strong predictive effect on organizational identification (*β*= 0.39, *p*< 0.01).

Thirdly, when both organizational identification and perceived CSR were included in the model, organizational identification significantly predicted both in-role (*β* = 0.22, *p* <0.01) and extra-role performance (*β* = 0.21, *p* <0.01). Concurrently, the predictive effects of perceived CSR decreased but remained significant, from *β* = 0.44 to 0.36 for in-role performance and from *β* = 0.40 to 0.32 for extra-role performance (*p* <0.01 for both; Model 3a and 3b). Therefore, organizational identification serves as a partial mediator in the relationship between perceived CSR and employee performance, providing support for Hypothesis 2.

**Comment 11: The explanation of the partial mediating effect of OI is unsatisfactory. It would be preferable to indicate what happens to the relevant coefficients when OI is included in Model 3.**

**My reply:**

I have rephrased the corresponding content to further highlight the meaning of "partial mediation", as follows (see blue font):

Thirdly, when both organizational identification and perceived CSR were included in the model, organizational identification significantly predicted both in-role (*β* = 0.22, *p* <0.01) and extra-role performance (*β* = 0.21, *p* <0.01). Concurrently, the predictive effects of perceived CSR decreased but remained significant, from *β* = 0.44 to 0.36 for in-role performance and from *β* = 0.40 to 0.32 for extra-role performance (*p* <0.01 for both; Model 3a and 3b). Therefore, organizational identification serves as a partial mediator in the relationship between perceived CSR and employee performance, providing support for Hypothesis 2.

**Comment 12: I would underline the fact that the interaction term in table 3 is significant at 5% level.
My reply**

When describing the significance of the interaction effect, I have added the expression "significant but weak", which equivalently emphasizes the magnitude of the interaction effect (see blue font below).

As shown in Table 3, the regression analysis revealed a significant but weak negative effect of the interaction term on organizational identification (*β=-*0.10, *p*<0.05) [51].

In addition, I argue that significance at the 0.05 level is still considered significant. If excessive emphasis is placed on the 0.05 threshold, it may instead lead to a counterproductive effect.

**Comment 13: DISCUSSION, IMPLICATIONS, LIMITATIONS AND CONCLUSIONS
These sections should be reconsidered in light of the requested revisions. At present, the claims are not, in the reviewer’s view, sufficiently well supported.**

**My reply:**

I have made substantial revisions to the entire paper, including adjustments to grammar and specific expressions, to enhance the clarity of the overall research 思路 and strengthen its logical coherence. I believe the revised paper has achieved a significant quality improvement and clearly demonstrates the research model and hypotheses.
Bibliography

**Comment 14: Podsakoff, P. M., MacKenzie, S. B., Lee, J. Y., & Podsakoff, N. P. (2003). Common method biases in behavioral research: a critical review of the literature and recommended remedies. Journal of applied psychology, 88(5), 879.**

**My reply:**

I have added this reference to the paper, as shown in the blue font below: First, Harman's single-factor test was conducted as an ex-post check[45]**.** It revealed four factors with eigenvalues exceeding 1, with the first factor explaining 32.72% of the total variance—below the 40% threshold—indicating no significant common method bias in the survey data[46].

I have added the following citation in the reference：

45.	Podsakoff PM, MacKenzie SB, Lee J-Y, Podsakoff NPJJoap. Common method biases in behavioral research: a critical review of the literature and recommended remedies. Journal of applied psychology. 2003;88(5):879.

**Finally, I would like to express my sincere gratitude to the editor and the two reviewers again. From your comments, I have learned a great deal about academic writing skills, and my research capabilities have been further enhanced!**
